# Exercise-based cardiac rehabilitation programmers for patients after transcatheter aortic valve implantation: A systematic review and meta-analysis

**DOI:** 10.1097/MD.0000000000034478

**Published:** 2023-07-28

**Authors:** Zhanjun Li, Wei Song, Na Yang, Yanyan Ding

**Affiliations:** a Department of Rehabilitation Medicine, Beijing Daxing District People’s Hospital, Beijing, China; b Department of Respiratory and Critical Care Medicine, Beijing Daxing District People’s Hospital, Beijing, China.

**Keywords:** aortic stenosis, cardiac rehabilitation, quality of life, transcatheter aortic valve implantation

## Abstract

Currently, transcatheter aortic valve implantation (TAVI) is presently a recognized treatment modality for patients with severe aortic stenosis who are often old, disabled, frail, and have low exercise capacity (ExCap). It is further expected from this therapy to improve quality of life by improving of the cardio function performance. The aim of this study is to evaluate the effect of exercise-based cardiac rehabilitation (CR) on patients after TAVI.

**Methods:**

PubMed, Embase, Cochrane Library, and Web of Science were searched from inception to December 10, 2022 for relevant studies that evaluated the effect of CR on patients after TAVI. The primary outcome was the improvement of 6-minute walked distance and Barthel index score after CR. The secondary outcomes included other parameters such as SF-12 scale, HADS score, Morse Fall Scale, Frailty-Index, ExCap, and FIM score. All statistical analyses were performed using the standard statistical procedures provided in Review Manager 5.2.

**Results:**

A total of 12 observational studies were identified, with 2365 participants. Pooled data indicated that CR programmers significantly improved the 6-minute walked distance (SMD 0.65; 95% confidence intervals [CI] 0.51–0.79) and Barthel index score (SMD 0.83; 95% CI 0.61–1.06). In addition, compared with admission, patients experienced significant improvement in SF-12 scale at CR discharge, with a pooled mean differences (MD) of 2.74 (95% CI 0.86–4.61) in physical component score and 2.76 (95% CI 0.59–4.93) in mental component score. Similar results were also observed in ExCap (MD 8.10 W; 95% CI 1.57 W–14.63 W) and FIM score (MD 11.0; 95% CI 6.22–15.78).

**Conclusions:**

Our analysis indicated that exercise-based CR programmers had significant effect on patients after TAVI in improving exercise tolerance and functional independence.

## 1. Introduction

Transcatheter aortic valve implantation (TAVI) has become the standard of care for patients with severe symptomatic aortic stenosis (AS) who are considered unsuitable for conventional surgery and may be a valid alternative to surgery in selected high-risk surgery patients. Since the first experience in 2002 >300,000 patients worldwide have undergone TAVI for severe AS.^[1–3]^ TAVI as a treatment option is increasingly recommended by surgeons and accepted by patients for more and more evidence supporting its benefits compared with traditional surgical treatment in patients with symptomatic severe AS who were unwilling or intolerant to surgical aortic valve replacement (sAVR). In addition, TAVI as a valid alternative option to sAVR in patients at high surgical risk was also been highly complimented.^[4–6]^ Furthermore, with improving and accumulating of surgeons’ experience, evolution of devices, and advancing techniques in the future, TAVI could be expand to more AS patients who must receive sAVR at present.^[7]^

Cardiac rehabilitation (CR) which based on exercise is recommended after cardiac surgery for improving exercise capacity (ExCap), exercise tolerance and functional independence with data also now showing its utility to improve quality of life (QoL), moderate frailty, and increase survival.^[8–12]^ Several studies have demonstrated the effectiveness of exercised-based residential after TAVI.^[9,13,14]^ Before our analysis, 2 meta-analyses which included only 7^[15]^ and 5 studies,^[11]^ respectively, evaluated the effectiveness of CR in patients after TAVI. However, considering the small number of studies and sample size, their result may have any risk of bias. In addition, previous studies always focused on the difference of main outcomes between CR admission and discharge in the same population. At present, at least 2 randomized controlled studies indicated no apparent difference between the control and intervention groups however at 3 or 6 months.^[16,17]^ Thus, we comprehensively searched and collected the studies that evaluated the effect of CR on patients after TAVI.

## 2. Methods

### 2.1. Search strategy and study selection

A systematic search of PubMed, Embase, Cochrane Library, and Web of Science from inception to December 10, 2022 was conducted for relevant studies using a search strategy developed by a medical information specialist that involved controlled vocabulary and keywords relating to transcatheter aortic valve implantation, CR, AS, or QoL were applied to the search strategy. The search strategy was limited to English language articles. All references were imported into Endnote, version X9 (Clarivate) for removal of duplicates. Manual screening of the references in the included articles was also conducted for a more comprehensive search. Two assessors independently screened the titles and abstracts of each study. When a relevant study was identified, its full text was obtained for further evaluation. The full text of related references was also obtained for review. The ethical approval of this study was not applicable.

### 2.2. Criteria for considering studies

We included studies if they met the following criteria: a. studies that evaluated the effect of CR on patients after TAVI; b. studies in which 6-minute walked distance (6-MWD) and Barthel index (BI) score or other indicators before and after CR were assessed.

Studies were excluded if they met the following criteria: a. experimental trial on animals or a nonhuman study, non-cohort studies; b. study population included patients with other diseases that would affect outcomes; c. study reported in the form of an abstract, letter, editorial, expert opinion, review, or case report; or d. lack of sufficient data or failure to meet the inclusion criteria.

### 2.3. Quality assessment and data extraction

Two reviewers assessed the quality of each study using the 9-star Newcastle-Ottawa Scale (NOS).^[18]^ The total NOS scores of each study were displayed in the characteristics table (Table 1). The scores were judged according to the 3 aspects of NOS of evaluation: selection, comparability, and outcome between the case group and control group. A study with a NOS score ≥ 6 is considered experiencing good quality. In addition, the risk of bias for each studies and the risk of bias across all studies were evaluated and shown with figures generated by RevMan 5.2 software.^[19]^

**Table 1:** The characteristics of each included studies for meta-analysis.

Study	Country	Study period	Sample size (n)	Male (%)	Age (year, mean ± SD)	BMI (kg/m^2^)	Interval time	CR duration	CR frequency	Main outcomes
Butter C (2018)	Germany	2008–2016	1017	44.5%	83.7 ± 3.6	24.7 ± 3.7	NR	3 weeks	6/week	6MWD, Barthel index
Eichler S (2017)	Germany	2013–2015	136	47.8%	80.6 ± 5.0	27.7 ± 4.2	17.7 ± 9.9 days	3 weeks	5/week	6-MWD, maximum work load, SF-12
Fauchère I (2014)	Switzerland	2008–2010	112	40%	79 ± 6	NR	NR	30 days	6/week	6-MWT, FIM, HADS score
Pressler A (2016)	Germany	2012–2014	30	54%	81 ± 6	26.9 ± 3.1	14 days	2 weeks	2/week, 3/week	6-MWT, KCCQ, SF-12
Rogers P (2018)	UK	2016–2017	27	44.4%	82.04 ± 4.8	27.70 ± 4.2	4 weeks	3 months	NR	6MWD, KCCQ, Nottingham EADL, Edmonton Frail Scale and HADS
Russo N (2014)	Italy	2008–2012	138	31.7%	83.7 ± 3.6	24.7 ± 3.7	NR	3 weeks	6/week	6MWD, Barthel index
Tarro Genta F (2017)	Italy	2010–2013	135	33%	82 ± 6	24 ± 4	3-week	3 weeks	6/week	6MWD, Barthel index, MFS
Tarro Genta F (2019)	Italy	2010–2014	90	34%	82.7 ± 4.9	24.8 ± 4.8	NR	3 weeks	6/week	6MWD, Barthel index, MFS
Tarro-Genta F (2015)	Italy	NR	110	28%	81.1 ± 4.6	24.1 ± 2.6	NR	3 weeks	NR	6MWD, Barthel index
Völler H (2015)	Germany	2009–2011	442	32%	69.94 ±•11.08	27.02 ± 4.66	NR	3 weeks	4–5/week	6MWD, ExCap, HADS
Yu Z (2021)	China	2019	69	60%	74.7 ± 8.1	23.3 ± 4.2	Before discharge after TAVI	1 month	NR	6-MWT, Barthel index, MMSE, HADS
Zanettini R (2014)	Italy	2009–2012	59	46%	83.5 ± 5.0	25.2 ± 4.5	NR	3 weeks	6/week	6MWD, Barthel index, EQ-VAS

6-MWT = 6 Minute Walking Test, EQ-VAS = EuroQoL visual analogue scale, ExCap = maximal exercise capacity, FIM = Functional independence measure, HADS = Hospital Anxiety and Depression Scale, KCCQ = the Kansas City Cardiomyopathy Questionnaire, MMSE = mini-mental state examination, NR = not report, SF-12 = 12-Item Short-Form.

Baseline characteristics and outcomes from the included studies were extracted using a standardized extraction form. Key study characteristics including country, study period, sample size, mean age, CR duration, CR frequency, interval time, and follow-up time were extracted. Data were extracted by one reviewer and then examined for accuracy and completeness by a second reviewer.

### 2.4. Data synthesis, statistical methods, and definitions

The data of comparable outcomes were combined-analyzed, using the standard statistical procedures provided in RevMan 5.2^[19]^ and Stata 12.0 (Stata Corp., College Station, TX). In this meta-analysis, the mean difference (MD) was sued before and after CR as the treatment effect, with a 95% confidence interval (CI). The heterogeneity between studies was evaluated by the chi-square-based Q statistical test,^[20]^ with *P*_*h*_ value and *I*^2^ statistic, ranging from 0% to 100 %, to quantify the effect of heterogeneity. *P*_*h*_ ≤ 0.10 was deemed to represent significant heterogeneity,^[21]^ and pooled estimates were estimated using a random-effect model (the DerSimonian and Laird method^[22]^). On the contrary, if statistical study heterogeneity was not observed (*P*_*h*_ > 0.10), a fixed effects model (the Mantel–Haenszel method^[23]^) was used. The effects of outcome measures were considered to be statistically significant if pooled MDs 95 % CI did not overlap with 0.

A sensitivity analysis was performed to examine the stability of the combined results and to identify the source of the heterogeneity. Finally, Funnel plot were prepared to detect publication bias. If the shape of the funnel plot revealed no obvious evidence of asymmetry, we considered that there was no obvious publication bias. All statistical analyses were performed using standard statistical procedures provided in RevMan 5.2 and Stata 12.0 (Stata Corp., College Station, TX).

This work has been reported in line with Preferred Reporting Items for Systematic Reviews and Meta-Analyses (PRISMA)^[24]^ and Assessing the methodological quality of systematic reviews (AMSTAR) Guidelines.^[25]^

## 3. Results

### 3.1. Included studies, study characteristics, and quality assessment

**Figure 1.**
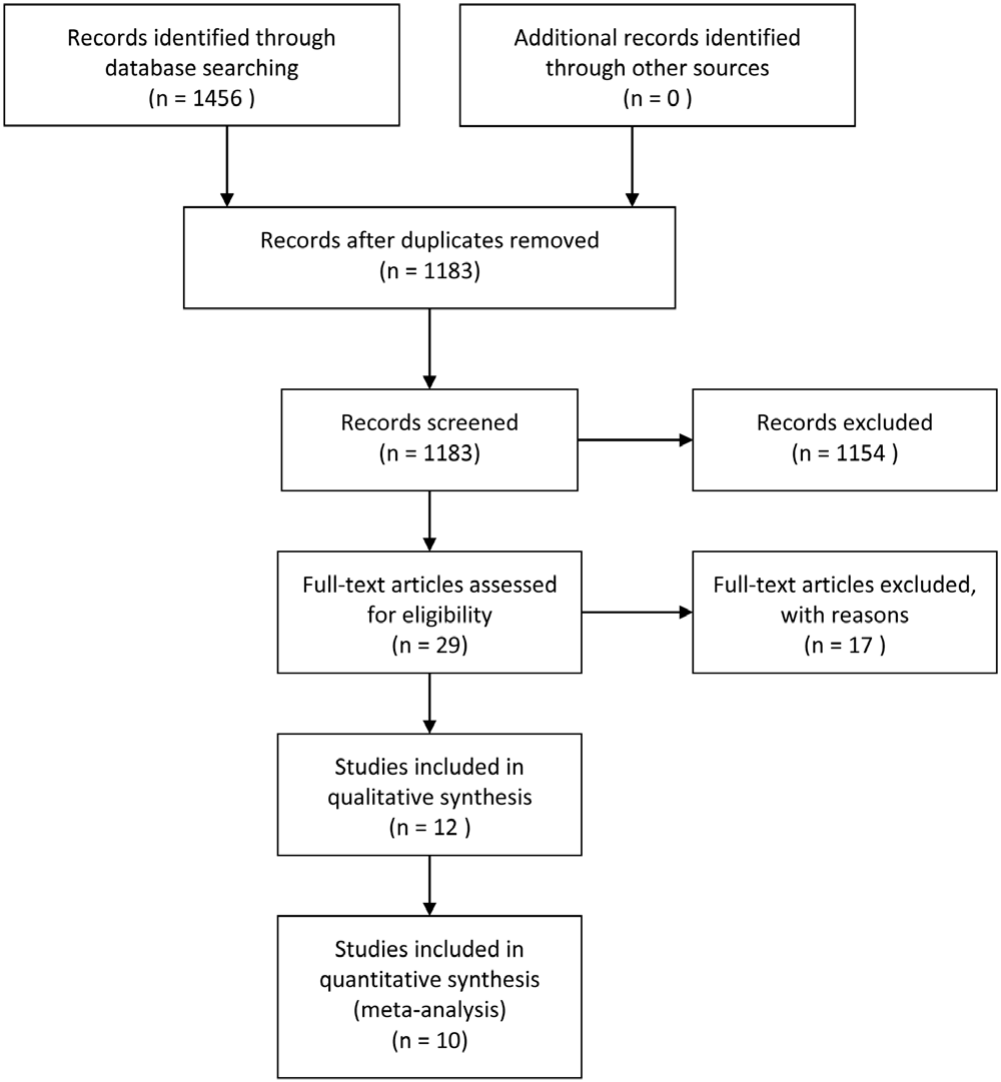
Flow diagram of literature search and selection of included studies for meta-analysis.

At the beginning of the search, a total of 1456 records of citations were obtained; 1183 of records were reviewed further after duplicates were removed. Via screening the titles and abstracts, 1154 studies were excluded preliminarily and then 29 studies were chosen to get full texts for further evaluation. After reading the full texts, 17 studies were excluded further as displayed in Figure 1. Eventually, 12 observational studies with 2365 participants were identified and included in this systematic review and meta-analysis. Of these studies, 4 were from Germany, 5 were from Italy, 1 from Switzerland, 1 from UK and 1 from China, respectively. The detailed search process and summary of studies are shown in the study flow diagram (Fig. 1). The other characteristics of each study are shown in Table 1.

The pooled baseline characteristics of the patients who received TAVI and the patients who received sAVR were provided in Table S1, Supplemental Digital Content, http://links.lww.com/MD/J383 (see Table S1, Supplemental Digital Content, http://links.lww.com/MD/J383, supplemental content, which illustrates the baseline characteristics of the patients who received TAVI and sAVR). As our pooled results, except mean aortic gradient, Euro-score, Karnofski index, Logistic EuroSCORE, CIRS-CI, and Clopidogrel use, the other of these comparative analyses of baseline characteristics (including age, gender, hypertension, diabetes, smoking, metabolic syndrome, coronary artery disease, previous MI, PCI, CABG, vascular disease, pulmonary disease, renal failure, left bundle branch block, implanted pacemaker, atrial fibrillation, previous TIA/stroke, BMI, e-GFR-CG, CKD at discharge, LVEF, NYHA class, hemoglobin at entry, and cardiovascular drugs) between patients who received TAVI and the patients who received sAVR reached conventional level of statistical significance.

According to our definitions, there was no poor quality studies included in this analysis. Additionally, risk-of-bias graphs were generated to further identify the risk of bias of the including studies. The risk of bias for each study was presented as percentages across all included studies, and the risk-of-bias item for each included study was displayed (see Figs. S1 and S2, Supplemental Digital Content, http://links.lww.com/MD/J384, supplemental content, which illustrates the risk-of-bias item for each included study). The risk-of-bias graphs indicated generally low risk of selection and comparability. In addition, all studies experienced low risk of bias in comparability and selection items except “ascertainment of exposure” item. A high risk of bias was mainly about follow-up issues in outcome items. Unclear risk of bias was mainly observed in “ascertainment of exposure,” “follow-up long enough for outcomes to occur” and “adequacy of follow-up of cohorts.”

### 3.2. CR effect for TAVI

#### 3.2.1. Effect on 6-MWD.

**Figure 2.**
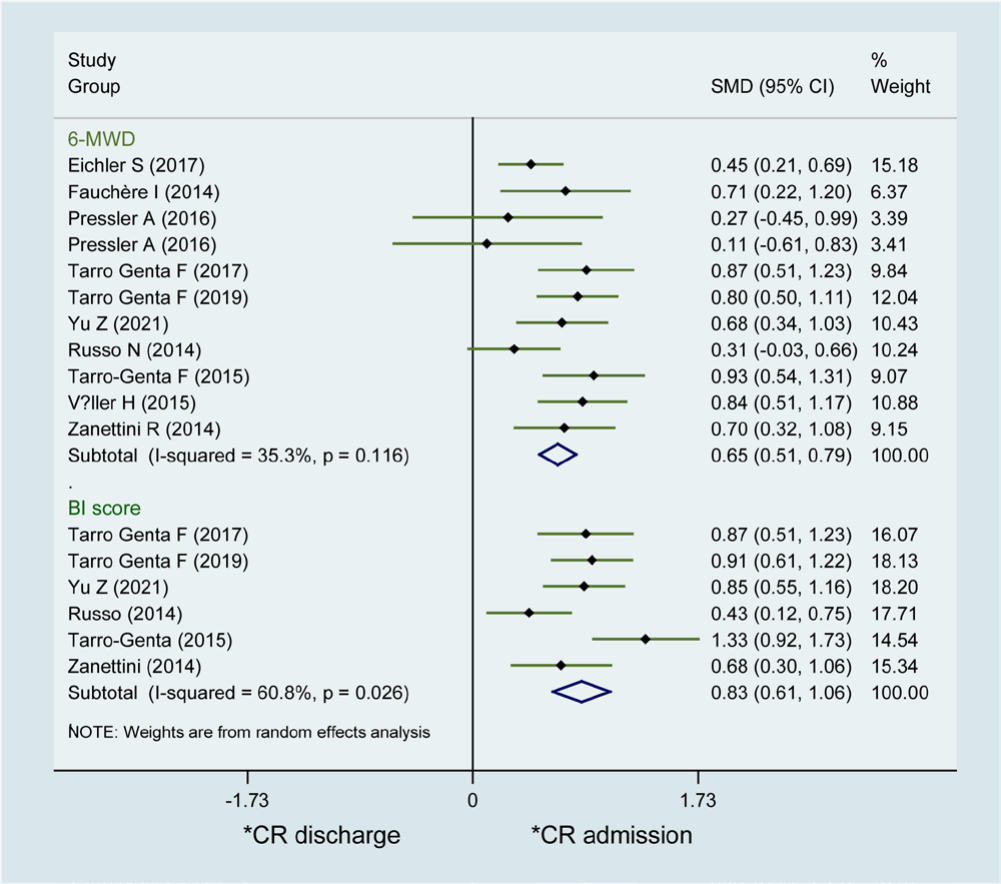
Forest plot of the cardiac rehabilitation effect on 6-minute walked distance and Barthel index score for patients received transcatheter aortic valve implantation.

A total of 11 studies compared the effect of CR for TAVI patients with 6-MWD. Pooled data indicated that CR programmers significantly improved the 6-MWD of patients after TAVI with a pooled SMD of 0.65 (95% CI 0.51–0.79; *I*^2^ = 35.3%) (Fig. 2). In addition, subgroup analysis also found similar results in TAVI patients receiving CR duration less than 1 month (SMD 0.63; 95% CI 0.52–0.74; *I*^2^ = 24%) and >1 month (SMD 0.76; 95% CI 0.46–1.06; *I*^2^ = 0%), respectively. We also explored the difference of CR frequency for the effect on 6-MWD. As a result, we found that CR programmers significantly improved the 6-MWD of TAVI patients who received CR frequency >6/week (SMD 0.62; 95% CI 0.48–0.76; *I*^2^ = 10%) as well as CR frequency <6/week (SMD 0.62; 95% CI 0.45–0.78; *I*^2^ = 5%).

#### 3.2.2. Effect on BI score.

We identified 6 studies that evaluated the effect of CR for TAVI patients using BI score. Our pooled result found that CR programmers also significantly improved BI score of patients after TAVI, with a pooled SMD of 0.83 (95% CI 0.61–1.06; *I*^2^ = 60.8%) (Fig. 2). In addition, we also conducted subgroup analyses according to CR duration and CR frequency TAVI patients received. As a result, similarly significant results were also found in both CR duration less than 1 month (SMD 0.81; 95% CI 0.51–0.92) and >1 month (SMD 0.65; 95% CI 0.42–0.87), respectively. BI score was also significantly improved by CR programmers in both CR frequency >6/week (SMD 0.80; 95% CI 0.46–0.94) as well as CR frequency <6/week (SMD 0.67; 95% CI 0.44–0.84).

### 3.3. CR effect on other parameters for TAVI

**Figure 3.**
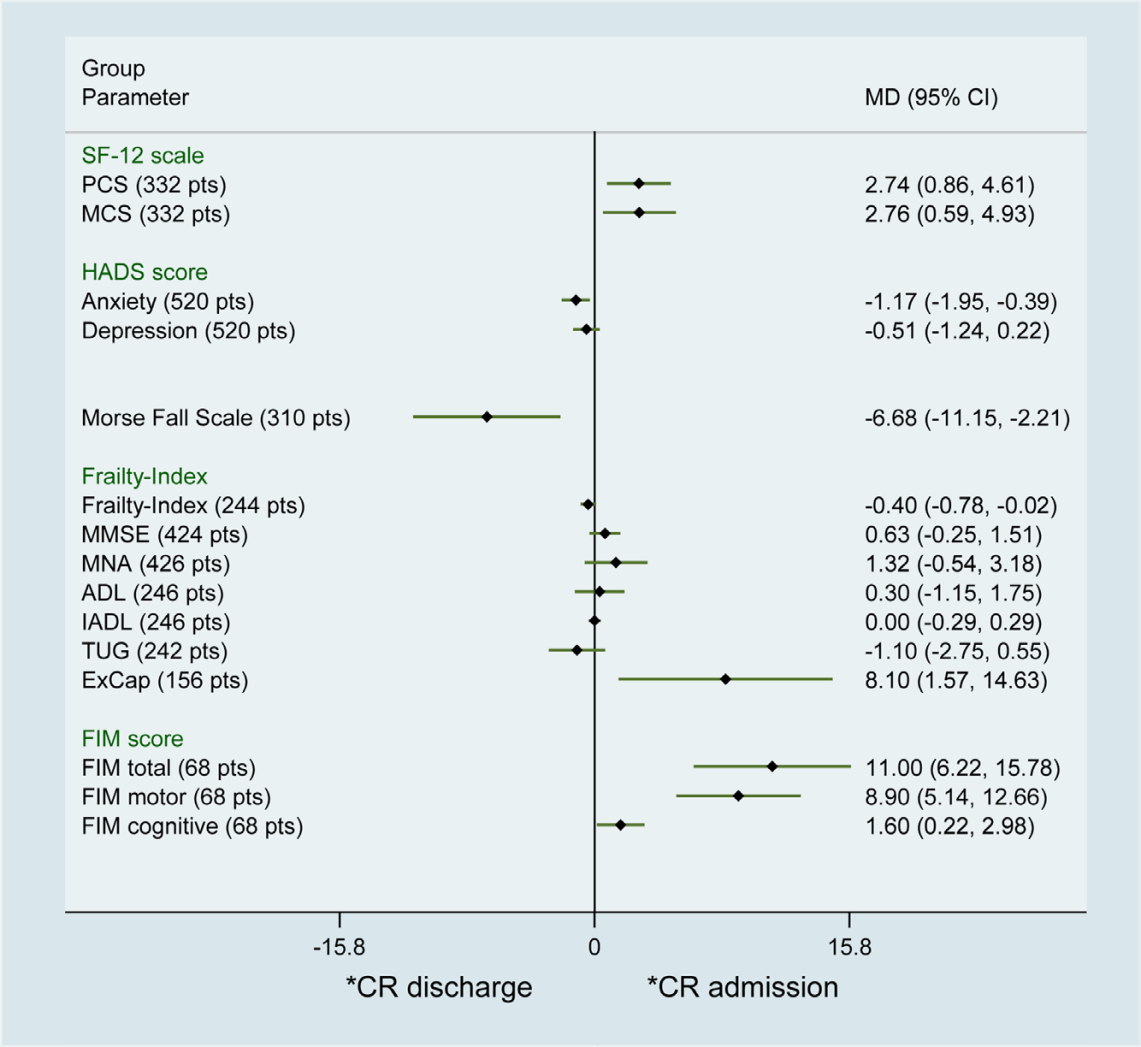
Pooled results of the cardiac rehabilitation effect on other parameters for patients received transcatheter aortic valve implantation.

In addition, we also assessed the effect of CR on SF-12 scale, HADS score, Morse Fall Scale, Frailty-Index, ExCap, and FIM score. Compared with admission, patients experienced significant improvement in SF-12 scale at CR discharge, with a pooled MDs of 2.74 (95% CI 0.86–4.61) in physical component score (PCS) and 2.76 (95% CI 0.59–4.93) in mental component score (MCS), respectively. Regarding to HADS score, significant result was also found anxiety score (MD ‐1.17; 95% CI ‐1.95 to ‐0.39) instead of depression score (MD ‐0.51; 95% CI ‐1.24 to 0.22). Significant result was observed in Morse Fall Scale (MD ‐6.68; 95% CI ‐11.15 to ‐2.21). Though significant result was observed in Frailty-Index (MD ‐0.40 point; 95% CI ‐0.78 to ‐0.02), no significance was found in the subgroup including MMSE, MNA, ADL, IADL, and TUG. Similar results were also observed in ExCap (MD 8.10 W; 95% CI 1.57 W–14.63 W; *P* = .02) and FIM score (MD 11.0; 95% CI 6.22–15.78), as well as FIM motor (MD 8.90; 95% CI 5.14–12.66) and FIM cognitive (MD 1.60; 95% CI 0.22–2.98) (Fig. 3).

### 3.4. CR effect on TAVI versus sAVR

**Figure 4.**
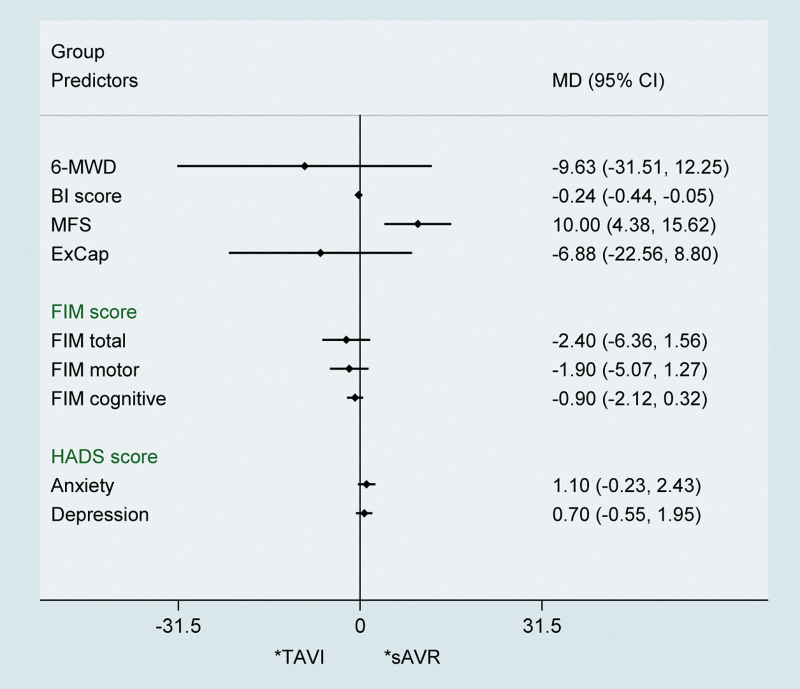
Pooled results of the cardiac rehabilitation effect on TAVI versus sAVR. sAVR = surgical aortic valve replacement, TAVI = transcatheter aortic valve implantation.

We compared the effect of CR programmers between TAVI and sAVR patients. As a result, it was indicated that CR programmers had equal effect on both TAVI and sAVR patients in 6-MWD (MD ‐9.63 m; 95% CI ‐31.51 to 12.25 m), FIM score [FIM total (MD ‐2.40; 95% CI ‐6.36 to 1.56), FIM motor (MD ‐1.90; 95% CI ‐5.07 to 1.27), FIM cognitive (MD ‐0.90; 95% CI ‐2.12 to 0.32)], HADS score [Anxiety (MD 1.10; 95% CI ‐0.23 to 2.43) and Depression (MD 0.70; 95% CI ‐0.55 to 1.95)] and ExCap (MD ‐6.88 W; 95% CI ‐22.56 to 8.80). However, significant difference was found in MFS (MD 10.00; 95% CI 4.38–15.62) and BI score (MD ‐0.24; 95% CI ‐0.44 to ‐0.05) (Fig. 4).

### 3.5. Publication bias and sensitivity analysis

**Figure 5.**
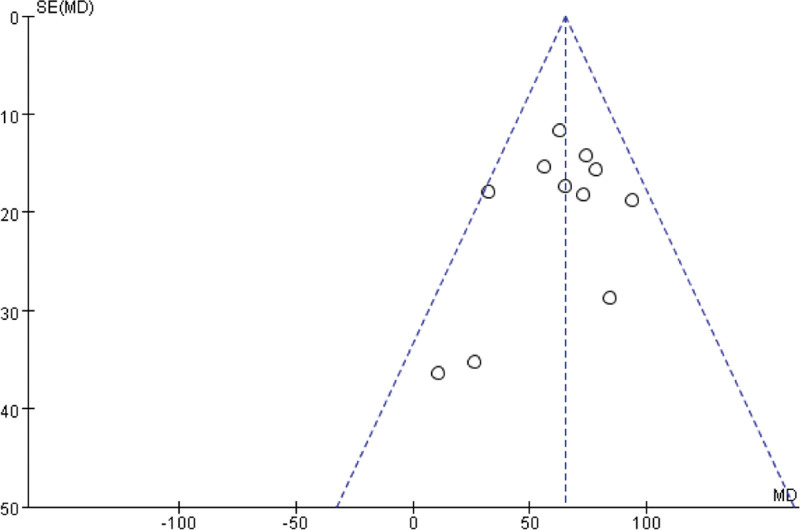
Funnel plot of the cardiac rehabilitation effect on 6-minute walked distance for patients received transcatheter aortic valve implantation.

Funnel plot was conducted for assessing the publication bias of included literatures and we could roughly assess the publication bias by seeing whether the shape was of any obvious asymmetry. According to Figure 5 showing, no clear evidence of publication bias was observed in the CR effect on 6-MWD for patients received TAVI. As shown in Figure S3, Supplemental Digital Content, http://links.lww.com/MD/J385 (see supplemental content, which illustrates sensitivity analysis by omitting each study from the list), no significant effect was observed from the exclusion of any single study, and the pooled results indicated good stability.

## 4. Discussion

The evolution of TAVI technology and techniques are contributing to the expansion of TAVI worldwide, with a rise in the number of older adults receiving TAVI who are disabled, frail, multimorbid, and enfeebled.^[7]^ Whereas CR is a logical consideration in this population, application has lagged. One review highlighted data showing that residential and ambulatory CR is safe and effective, with metrics of reduced disability and frailty, improved ExCap, QOL, and short-term survival.^[26]^ Several studies have demonstrated the effectiveness of exercised-based residential after TAVI.^[9,14,27]^

As far as we know, before our analysis, there were 2 meta-analyses which included only 7^[15]^ and 5 studies,^[11]^ respectively evaluating the effectiveness of CR in patients after TAVI mainly focused on 6-WMD and BI score. The previous results were lack of comprehensiveness for the effectiveness of CR and were failed to contain many parameters such as SF-12 scale, HADS score, Morse Fall Scale, Frailty-Index, ExCap, and FIM score. In addition, previous studies always focused on the difference of main outcomes between CR admission and discharge in the same population. At present, at least 2 randomized controlled studies indicated no apparent difference between the control and intervention groups however at 3 or 6 months.^[16,17]^ Thus, we comprehensively searched and collected the studies that evaluated the effect of CR on patients after TAVI based on 12 observational studies with 2365 participants.

Our pooled analyses indicated that CR programmers significantly improved the 6-MWD (MD 65 m; 95% CI 55 m–75 m) and BI score (MD 14; 95% CI 8–19). The results were consistent with the report from Oz A (2022).^[15]^ In addition, compared with admission, patients experienced significant improvement in SF-12 scale at CR discharge, with a pooled MDs of 2.74 (95% CI 0.86–4.61; *P* = .004) in physical component score and 2.76 (95% CI 0.59–4.93; *P* = .01) in mental component score. Similar results were also observed in ExCap (MD 8.10 W; 95% CI 1.57 W–14.63 W; *P* = .02) and FIM score (MD 11.0; 95% CI 6.22–15.78; *P* < .0001). There were significant benefits in QoL derived for both TAVR inpatient CR (EuroQol visual analogic scale from 54 ± 14 to 75 ± 11, *P* < .001).^[28]^ Compared with admission, patients experienced significant improvement in SF-12 scale at CR discharge, with a pooled MDs of 2.74 (95% CI 0.86–4.61; *P* = .004) in PCS and 2.76 (95% CI 0.59–4.93; *P* = .01) in MCS, respectively. Similarly, the results were also demonstrated by Eichler S (2017) in inpatient CR (35.9 ± 8.8 to 38.3 ± 8.3, *P* = .001 in PCS and 47.3 ± 10.6 to 50.7 ± 10.0, *P* = .003 in MCS, respectively)^[29]^ and by Pressler A (2016) in outpatient CR (39.5 ± 10.0 improved to 45.9 ± 8.9, *P* < .05 in PCS).^[17]^ The safety of CR programs was also demonstrated and there were no recorded adverse events associated with the intervention of CR however at the follow-up of 3 or 6 months.^[16]^ In addition, a program of rehabilitation after TAVI has the potential to reduce mortality. Mortality at 6 months was lower for patients receiving rehabilitation compared with those who had not (OR 0.49; 95% CI 0.25–0.94; *P* = .032).^[9]^ Sub-analysis showed the benefit of CR (OR 0.31; 95% CI 0.14–0.71, *P* = .006), but not geriatric rehabilitation (OR 0.83; 95% CI 0.37–1.85, *P* = .65).^[9]^

Excise-based CR programs may not only have effect on the functional and psychocognitive recovery as well as QoL improvement for patients after TAVI, but also benefit to survival of patients after TAVI. It was proved that 6-MWT, Morse Fall Scale, and BI score, which could be improved by CR programs, were prognostic factors for survival at 3 years.^[14]^ Similarly, more improvement of 6-MWT, Morse Fall Scale, and BI score was also confirmed in survivors than nonsurvivors after TAVI at follow-up.^[14]^

There existed several limitations in our work. First, due to lack of patient-level data, we could not perform additional subgroup analyses according to other baseline characteristics. Second, the majority of our included studies were designed as retrospective studies, which could be influenced by many factors as its natural attribute. Thus, the level of evidence of our results may be low. Third, there were 3 prospective studies in our identified studies.^[9,16,17]^ Though our pooled results affirmed the effect CR on patients after TAVI, inconsistent results were showed in the studies from Pressler A (2016)^[17]^ and Rogers P (2018).^[16]^ Fourth, previous studies as well as meta-analyses always studied the effect of CR on patients after TAVI focusing on the difference of main outcomes between CR admission and discharge in the same population and found affirmative conclusion. However, the effect of CR on patients after TAVI was controversial in prospective studies. For example, 1 randomized pilot trial that compared the usual care group and exercise-based CR group demonstrated that exercise training in patients after TAVI was safe and highly effective with respect to improvements in ExCap, muscular strength, and QoL 6-MWD.^[17]^ However, another randomized controlled study indicated no apparent difference between the control and intervention groups however at 3 or 6 months.^[16]^ Fifth, we failed to evaluate the influence of interval time between operation of TAVI to CR beginning, CR duration and frequency on the effect of CR. There was study that indicated that both the days from implantation to CR admission (HR 1.05; 95% CI 1.00–1.10; *P* = .043) and length of CR stay (HR 1.048; 95% CI 1.02–1.07; *P* = .000) were prognostic factors for survival of patients after TAVI at 3 years.^[14]^ Finally, the follow-up time of our included studies was 3 to 6 months. Thus, the long term effect of CR on the patients after TAVI could not been evaluated. Zanettini R (2014) evaluated the effect of CR on patients after TAVI with a midterm follow-up of 540 days (range: 192–738 days) and came to a conclusion that during a rehabilitation programme, based on a multidimensional assessment and intervention, most patients showed significant improvement in functional status, QoL, and autonomy, which remained stable in the majority of subjects during midterm follow-up.^[28]^

Considering our analysis results and limitations, there are several suggestions for future researches. (1) A study with a randomized controlled or prospective design should been conducted to clear the different outcomes or survival between patients receiving excise-based CR program and patients receiving usual care or no CR program. (2) Propensity score-matched (PSM) studies with enough sample size are needed to demonstrate the efficacy of CR for patients after TAVI. (3) In addition, future studies should clear which group of patients in which phase after operation benefit more in CR programmers. Enough detailed evaluation of variables, such as frailty, nutritional status, depression, or musculoskeletal status, may have provided further insight into the benefits of the different components of the rehabilitation programs, which should be focused in future prospective studies. (4) Future research should increase the intensity and duration of CR, shorten the time to CR start, to clear the influence of these factors on effect of CR. (5) More detailed grouping or subgroup analysis according to different characteristics of patients is needed to clear which patients may benefit more from CR. (6) With consideration of follow-up <6 months in present studies, future researches should lengthen the follow-up time in order to clear the difference or effectiveness on long-term outcomes of CR for patients after TAVI.

## 5. Conclusions

In conclusion, our analysis indicated that exercise-based CR programmers had significant effect on patients after TAVI in improving exercise tolerance and functional independence.

## Author contributions

**Conceptualization:** Zhanjun Li, Wei Song.

**Data curation:** Zhanjun Li, Wei Song.

**Methodology:** Zhanjun Li, Wei Song, Na Yang, Yanyan Ding.

**Software:** Zhanjun Li, Na Yang, Yanyan Ding.

**Writing – original draft:** Zhanjun Li, Wei Song, Na Yang.

**Writing – review & editing:** Zhanjun Li, Wei Song, Na Yang, Yanyan Ding.

## Supplementary Material






