# Supplementary material for: Exercise-based cardiac rehabilitation programmers for patients after transcatheter aortic valve implantation: A systematic review and meta-analysis
Source: Medicine (Baltimore). 2023 Jul 28;102(30):e34478. doi: 10.1097/MD.0000000000034478 (PMC10378889; doi:10.1097/MD.0000000000034478)
Supplement: Supplementary file 1 [file medi-102-e34478-s001.pdf]

Table S1. The baseline characteristics of the patients who received TAVI and sAVR.

Characteristics	Total (n = 847)	TAVI (n = 253)	sAVR (n = 594)	*P* value
Age (years) , mean ± SD	79.2 ± 1.6	80.3 ± 1.6	78.2 ± 1.5	0.06
Male, n	430	189	241	0.22
Hypertension, n	358	154	203	0.49
Diabetes, n	191	60	131	0.11
Current smoker, n	6	3	3	0.23
Smoking history, n	20	7	13	0.47
Metabolic Syndrome, n	112	49	63	0.13
Coronary artery disease, n	328	99	229	0.06
Previous MI, n	26	10	16	0.37
Previous PCI, n	112	46	66	0.17
Previous CABG, n	10	6	4	0.08
Vascular disease, n	35	21	14	0.34
Pulmonary disease, n	80	29	51	0.33
Renal failure, n	39	19	20	0.14
LBBB, n	94	30	64	0.07
Implanted pacemaker, n	52	21	31	0.32
Atrial fibrillation, n	110	30	80	0.781
Previous TIA/stroke, n	28	16	12	0.06
BMI (kg/m2) , mean ± SD	27.7 ± 5.9	28.2 ± 5.4	27.6 ± 6.1	0.14
e-GFR-CG (ml/min), mean ± SD	43 ± 14	39 ± 12	46 ± 14	0.12
CKD at discharge (e-GFR <60ml/min), n	263	94	169	0.11
LVEF(%), mean ± SD	56.7 ± 9.8	55.9 ± 11.3	57.2 ± 8.5	0.55
Mean aortic gradient (mmHg), mean ± SD	12.8 ± 5.6	9.3 ± 3.6	15.9 ± 5.2	< 0.001
NYHA class, mean ± SD	2.8 ± 0.8	2.9 ± 0.8	2.7 ± 0.8	0.22
Euro-score, mean ± SD	15.6 ± 12.1	24.8 ± 13.6	11.6 ± 8.9	< 0.001
Karnofski index, mean ± SD	1.8 ± 0.4	1.6 ± 0.5	1. 9 ± 0.3	< 0.001
Logistic EuroSCORE, mean ± SD	14.4 ± 10.6	21.4 ± 10.7	7.9 ± 5	< 0.001
CIRS-CI, mean ± SD	4.1 ± 1.7	4.8 ± 1.5	3.4 ± 1.5	< 0.001
Haemoglobin at entry (g/dL), mean ± SD	10.2 ± 0.9	10.3 ± 0.9	10.2 ± 1.0	0.33
Stay in CR (days) , mean ± SD	16.4 ± 3.9	16.6 ± 4.7	16.1 ± 2.9	0.58
Time implant-rehab (days) , mean ± SD	13.7 ± 11.7	13.3 ± 12.5	14.2 ± 10.8	0.55

Aspirin, n	218	92	126	0.31
Clopidogrel, n	120	115	5	< 0.0001
Warfarin, n	143	103	40	0.06
ß-blockers, n	190	111	79	0.78
Calcium antagonist, n	29	20	9	0.11
Amiodarone, n	47	13	34	0.01
ACE-I, n	137	65	72	0.07
AT-II ant, n	29	18	11	0.31
Diuretics, n	213	119	101	0.36
Statins, n	110	60	50	0.18

CABG, coronary artery bypass graft; MI, myocardial infarction; PCI, percutaneous coronary intervention; LBBB, Left bundle branch block; BMI, body mass index; LVEF, Left ventricular ejection fraction; NYHA, New York Heart Association; ACE-I, angiotensin-converting enzyme inhibitor; AT-II ant, angiotensin II receptor antagonist; CIRS-CI, Cumulative Illness Rated State-Comorbidity Index.
