# Supplementary material for: Exercise-based cardiac rehabilitation programmers for patients after transcatheter aortic valve implantation: A systematic review and meta-analysis
Source: Medicine (Baltimore). 2023 Jul 28;102(30):e34478. doi: 10.1097/MD.0000000000034478 (PMC10378889; doi:10.1097/MD.0000000000034478)
Supplement: Supplementary file 2 [file medi-102-e34478-s002.pdf]

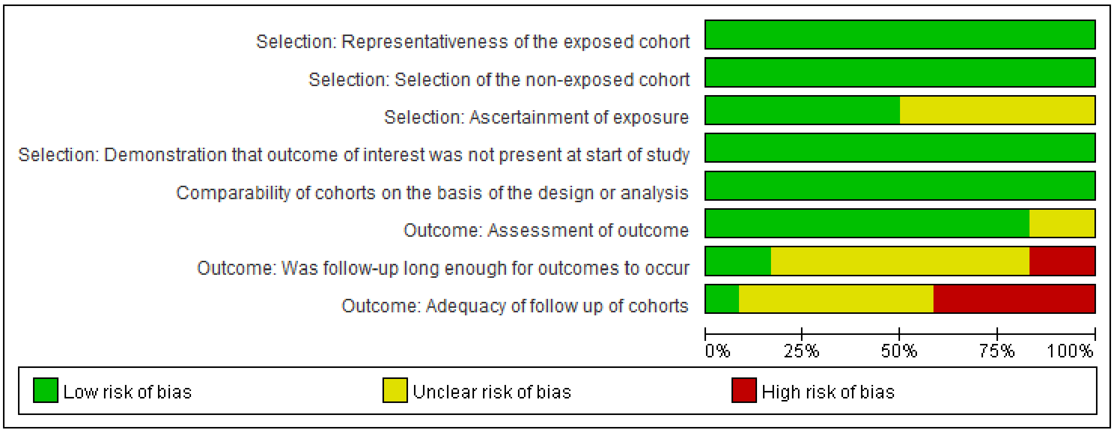

Figure S1. Risk of bias graph: review authors' judgements about each risk of bias item presented as percentages across all included studies.

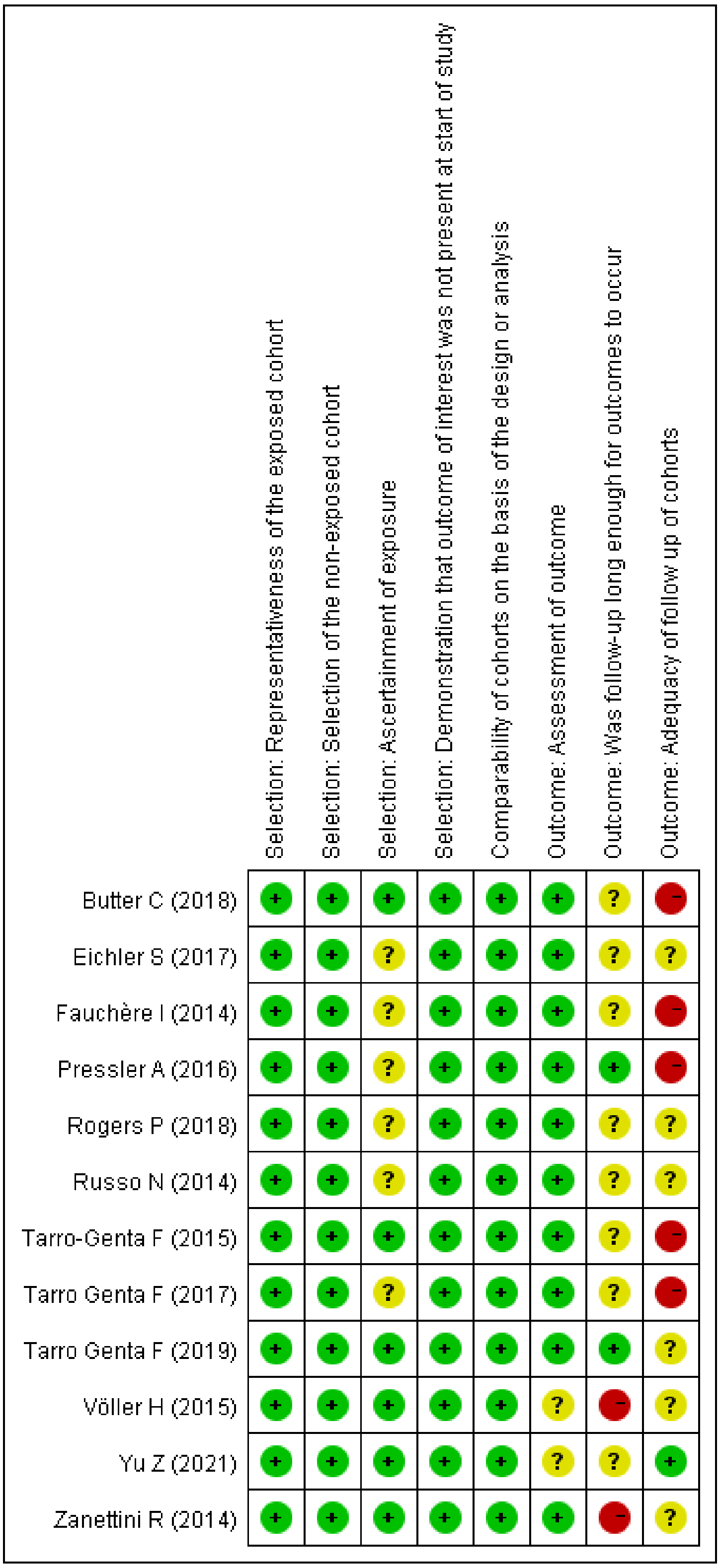

Figure S2. Risk of bias summary: review authors' judgements about each risk of bias item for each included study.
