# Supplementary figures and images for: Exercise-based cardiac rehabilitation programmers for patients after transcatheter aortic valve implantation: A systematic review and meta-analysis
Source: Medicine (Baltimore). 2023 Jul 28;102(30):e34478. doi: 10.1097/MD.0000000000034478 (PMC10378889; doi:10.1097/MD.0000000000034478)

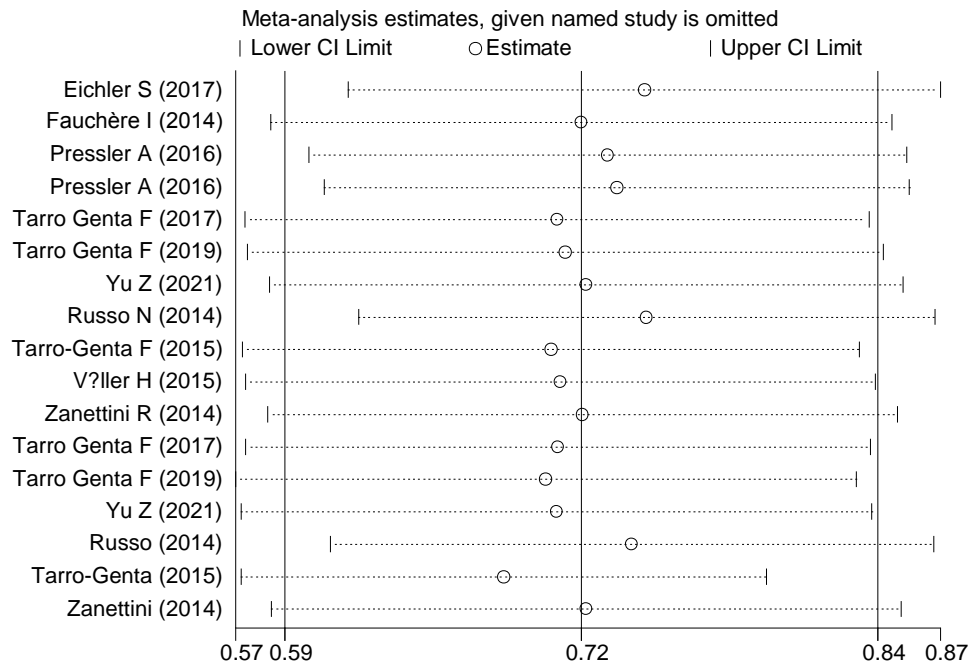

**Figure S3.** Sensitivity analysis by omitting each study from the list.

Supplement: Supplementary file 3 [file medi-102-e34478-s003.pdf]
